# Mini-Review: Human Microbiome and Rheumatic Diseases

**DOI:** 10.3389/fcimb.2020.491160

**Published:** 2020-11-10

**Authors:** Meltem Vural, Benoit Gilbert, Işıl Üstün, Sibel Caglar, Axel Finckh

**Affiliations:** ^1^Physical Medicine and Rehabilitation Clinic, University of Health Sciences, Bakırkoy Dr. Sadi Konuk Training Hospital, Istanbul, Turkey; ^2^Rheumatology Division, Department of Medicine, Geneva University Hospital (HUG), Geneva, Switzerland

**Keywords:** microbiome, rheumatic diseases, etiopathogenesis, spondylarthritis, rheumatoid arthritis

## Abstract

Rheumatoid arthritis and spondyloarthropathy are the most common inflammatory rheumatic diseases. As the human microbiome is involved in the immune homeostasis, it has the potential to be a key factor in the development of autoimmune diseases and rheumatic diseases. In this article, we review the role of various human microbiota on the pathogenesis of rheumatic diseases, focusing on spondylarthritis and rheumatoid arthritis.

## Introduction

Evidence from animal and human translational research suggests an important function of the microbiome in modulating the host’s immunity (Longman and Littman, 2015). Although disruptions in local microbiome composition (dysbiosis) can result in a systemic inflammatory response (Littman and Pamer, 2011; Manasson and Scher, 2015; Scher et al., 2016), the development of an inflammatory disease would require a disturbance of the complex network of interactions between the microbiome, genetic factors and the environment (Kim et al., 2016; Picchianti-Diamanti et al., 2018).

Rheumatic musculoskeletal diseases (RMDs) are the second cause of years lived with disability worldwide (Vos et al., 2015). Rheumatoid Arthritis (RA), is the most prevalent chronic auto-immune disease. It leads to peripheral joint destruction and extra-articular manifestations, usually sparring distal interphalangeal and spinal joints (Majithia and Geraci, 2007). RA incidence rate is still rising (Safiri et al., 2019), and prevalence approximates 0.5% to 1% in the European and North American population (Cooper and Stroehla, 2003). Diagnosis is mainly clinical, but identification of auto-antibodies, such as rheumatoid factor or anti-citrullinated-peptides antibodies (ACPA) has a high positive predictive value (Majithia and Geraci, 2007). Unlike RA, spondylarthritis (SpA) refers to a group of clinically and genetically related disorders for which no specific autoantibody has been identified, which explains why the SpA are not considered an autoimmune disease, but classified as an “immune-mediated inflammatory diseases”. SpA includes ankylosing spondylitis (AS), psoriatic arthritis (PsA), juvenile SpA, reactive arthritis (ReA), and inflammatory bowel disease (IBD)-related arthritis (Asquith et al., 2014). SpA is considered to have a more heterogeneous clinical spectrum, usually involving spinal joints, in association with peripheral manifestations, distinct from RA symptomatology.

Recent advances in sequencing technologies have greatly improved systematic and comprehensive investigations of the human microbiome (Costello et al., 2015a). DNA-based taxonomy allows identification at the species level, while microbial metagenomics and metatranscriptomics respectively inform about functional capacity and actively transcribed genes in the microbiome (Goodrich et al., 2014; Sharpton, 2014; Hiergeist et al., 2015; Coit and Sawalha, 2016). The evolutionary process of co-adaptation, as well as the direct and indirect crosstalk between the intestinal microbiome and the host immune system is now increasingly appreciated (Scher et al., 2016; Caminer et al., 2017). Thanks to the use of these novel microbiologic techniques, the human microbiome has increasingly been recognized as a potential key factor in the development of autoimmune diseases (Manasson and Scher, 2015; Scher et al., 2016) and RMDs.

Herein, we review the role of the microbiome in specific human rheumatic diseases focusing on spondylarthritis and rheumatoid arthritis.

## Human Microbiome in Spondylarthritis

With a relatively larger participation of genetics in the disease etiopathogenesis, spondylarthritis also remarkably depends on the role of pro-inflammatory Th17 cells and its signature cytokine, IL-17 (Mielants et al., 1995; Scher et al., 2016). Hence, intestinal microbiota, gut inflammation and SpA are considered to have an epidemiological link, as supported by the demonstrated role of infectious diarrhea in the pathogenesis of ReA, and the association between IBD and AS and PsA (Mielants et al., 1995; Arvikar and Fisher, 2011; Li W.-Q. et al., 2013). The Human Leukocyte Antigen B27 (HLA B27) is a MHC Class I molecule. HLA B27 association with SpA is the most relevant MHC association in human diseases. There are several hypothesis for the role of HLA B27 molecule in SpA: 1) arthritogenic peptide recognized by HLA B27, 2) HLA-B27 protein misfolding resulting in intracellular stress and 3) innate immune recognition of aberrant HLA-B27 (Brown, 2014). The binding of HLA-B27 homodimers to KIR3DL2-positive cells can stimulate IL-17 production, and CD4 T cells positive for KIR3DL2 in the synovial fluid of patients with spondylarthritis demonstrate increased IL-17 secretion, IL-23 receptor expression and production of inflammatory cytokins (Chen L. et al., 2016).

Animal studies have described spontaneous inflammatory SpA-like disease in transgenic rats expressing HLA-B27, which have a phenotype characterized by sacroiliitis, peripheral arthritis, psoriasiform skin inflammation and colitis (Hammer et al., 1990; Taurog et al., 1994). The disease predisposition associated with this allele has been linked to HLA-B27-mediated alterations in the intestinal microbiome by a combination of HLA-B27 misfolding and activation of intestinal and circulating Th17 cells, in an IL-23–dependent manner (DeLay et al., 2009; Rosenbaum and Davey, 2011; Glatigny et al., 2012). Data from other animal models have also indicated the role of gut bacteria in SpA inflammation, such as spontaneous development of progressive enthesitis and ankylosis triggered by commensals in the ANKENT mouse model (Šinkorová et al., 2008). ANKENT mice having HLA B27 spontaneously develop a progressive ankylosing enthesopathy of ankle and tarsal joints (Weinreich et al., 1995). SKG is a mouse strain that develops chronic autoimmune arthritis similar to RA (Sakaguchi et al., 2003). Also the development of SpA-like disease after a systemic injection of β-glucan through downstream production of IL-23–dependent IL-17/IL-2270 in the SKG mouse model reinforces this issue (Ruutu et al., 2012; Sherlock et al., 2012). A link between IL23R polymorphisms and susceptibility to psoriasis, PsA and AS has also been suggested (Liu et al., 2008).

Hence, a disrupted gut environment is hypothesized to be associated with dysregulated immune response and/or altered dendritic cell function in spondyloarthropathy (Stoll, 2015). There is substantial evidence regarding the important role of the microbiota in SpA through multiple overlapping mechanisms, including alteration of intestinal permeability (possibly determined by genetic factors such as HLA-B27 expression), decreased production of anti-inflammatory metabolic products, activation of intestinal immunity, and migration of microbial products to the peripheral joints with subsequent local immune activation (Rosenbaum and Davey, 2011; Lin et al., 2014; Stoll, 2015; Picchianti-Diamanti et al., 2018).

In a study with 9 AS patients and 9 healthy controls, terminal ileum biopsy specimens were generated using 16S ribosomal RNA gene sequences and analysis techniques in SpA patients, gut microbial analyses reported higher abundance of *Lachnospiraceae*, *Ruminococcaceae*, *Rikenellaceae*, *Porphyromonadaceae*, and *Bacteroidaceae*, and lower abundance of *Veillonellaceae* and *Prevotellaceae* compared to healthy controls (Costello et al., 2015b). Decreased numbers of *Firmicutes*, major phyla of gut commensals, particularly *Faecalibacterium prausnitzii* and *Clostridium leptum* species were also reported in SpA and IBD patients and considered an important link between SpA and gut inflammation (Gill et al., 2015).

### Ankylosing Spondylitis

A direct link between systemic inflammation and gut dysfunction in humans has been clearly identified only in AS, including several alterations of the ileum architecture, disruption of the basal membrane with compromised epithelial cell permeability, hyperplasia of goblet cells and activation of Paneth cells, producing high levels of adenosine monophosphate and proinflammatory cytokines, such as IL23 (Ciccia et al., 2010; Ciccia et al., 2012; Ciccia et al., 2016, p. 23; Picchianti-Diamanti et al., 2018).

The microbial community of terminal ileal biopsies from AS patients and healthy controls demonstrated the presence of an intestinal dysbiosis, with a more diverse terminal ileum microbiota signature in AS patients compared to healthy controls (Costello et al., 2015b). The dysbiosis in the AS gut was indicated to be driven by higher abundance of five families of bacteria *Lachnospiraceae*, *Ruminococcaceae*, *Rikenellaceae*, *Porphyromonadaceae* and *Bacteroidaceae* and a decrease in abundance of two families of *Veilonellaceae*, without an overall alteration in microbial load or an overgrowth or dominance of a specific microbe (Costello et al., 2015b). A higher prevalence of sulfate-reducing bacteria was also reported in patients with AS. The sulfate‐reducing bacteria can reduce inorganic sulfate to hydrogen sulfide. They are hydrogen scavengers. Higher prevalence of these bacteria may demonstrate gut inflammation (Stebbings, 2002). In children with enthesitis-related arthritis (according to the International League of Associations for Rheumatology criteria, juvenile idiopathic arthritis subtype), the relative abundance of *F. prausnitzii* was reported to be significantly lower as compared to healthy controls. This finding has also been reported in patients with IBD (Thorkildsen et al., 2013; Stoll et al., 2014).

### Psoriatic Arthritis

The overexpression of IL9 and the production of Th9, Th17, and Th22 responses produce subclinical gut inflammation in PSA patients (Picchianti-Diamanti et al., 2018). Furthermore, gut-derived Th9 cells have the ability to migrate and enter synovial tissues, before initiating a positive autocrine loop and local inflammation created *via* IL9 and IL9 receptor expression by gut-activated Paneth cells (Ciccia et al., 2016).

Analysis of the gut microbiota with high-throughput 16S rRNA pyrosequencing method in a cohort of PsA and psoriasis patients demonstrated a decrease in taxonomic diversity due to lower abundances of several taxa compared to the microbiota of healthy controls (Scher et al., 2015), including decreased *Coprococcus* in both psoriasis and PsA patients, while significantly lower levels of *Akkermansia* and *Ruminococcus* only in the PsA group (Scher et al., 2015; Scher et al., 2016). This decline in *Ruminococcus* and *Akkermansia* specific to PsA has been interpreted as a chronological loss of diversity that may potentially associate with the natural history of disease (Scher et al., 2015; Scher et al., 2016). Interestingly, a similar reduction of *Ruminococcaceae* family and *Akkermansia* genus has also been reported in patients with IBD (Willing et al., 2010; Scher et al., 2016). In addition, the decreased abundance of *Akkermansia* in PsA contrasts to that of juvenile SpA, emphasizing the likelihood of distinct microbes in the etiology of these diseases (Gill et al., 2015). A hallmark of intestinal dysbiosis in PsA patients is considered to be the loss of commensals responsible for mucus degradation and small and medium chain fatty acid production. This situation suggests a relation between microbiome perturbations, mucosal integrity and the dissemination of systemic inflammation (Gill et al., 2015; Coit and Sawalha, 2016; Scher et al., 2016).

Data from recent 16S rRNA sequencing studies have also indicated significant differences in cutaneous microbiota between psoriasis patients and controls, and between psoriatic lesions and control skin samples, with greater abundance of *Firmicutes* and lesser abundance of phylum *Actinobacteria*, *Staphylococci*, and *Propionibacteria* in cases and in affected skin (Gao et al., 2008; Fahlén et al., 2012; Costello et al., 2015a). Large shifts of normal phyla of the human skin and reduction of *Propionibacteria* may be the cause or result of drastic changes in diseased skin. It still remains unknown ıf it is a potential biomarker to predict who eventually develop psoriatic arthritis (Coit and Sawalha, 2016).

### Juvenile SpA

Patients with a specific form of juvenile SpA, classified as enthesitis-related arthritis (ERA), were reported to exhibit decreased abundance of *C. leptum*, similar to AS patients (Stebbings, 2002; Stoll et al., 2014). Furthermore, a decrease in *F. prausnitzii* (another member of the *Clostridales* family) and in cellular immune response to *Salmonella typhimurium* were also reported in patients with juvenile SpA compared to healthy controls (Singh et al., 2011; Gill et al., 2015).

## Human Microbiome in Rheumatoid Arthritis (RA)

The etiopathogenesis of RA is partially understood and believed to result from a multi-step process. Various environmental factors would insidiously initiate a pathological activation of the immune system in genetically susceptible individuals (Klareskog et al., 2006). This aberrant immune activation would result in asymptomatic presence of autoantibodies, such as rheumatoid factor or anti-citrullinated-peptide antibodies (ACPA) (Klareskog et al., 2006). A subsequent pauci-symptomatic or “pre-clinical” phase usually precedes the clinically apparent evolution toward classifiable RA (Klareskog et al., 2006).

This “immune onset” of the disease is thought to be initiated in extra-articular tissues before localizing in the synovium (Holers et al., 2018). Hence, the mucosal origins hypotheses of RA (Li S et al., 2013) suspect a role of microbiota in dysregulating immune response. Proposed mechanisms (Li S et al., 2013) linking microbes to autoimmunity include: - 1) the microbial generation of neo-autoantigens (such as citrullinated peptides) targeted by the immune system; - 2) a loss of tolerance of the immune system against normal self-antigens *via* molecular mimicry [e.g. rheumatic fever (Cunningham, 2000)]; - 3) a bystander activation of the immune system *via* a non-specific activation of auto-reactive T cells by microbes or microbial super-antigens.

### The Oral Microbiota and RA

Periodontitis is clearly associated with established RA (Potikuri et al., 2012; Scher et al., 2012; Eriksson et al., 2019), but the causal relationship has only been studied in a very limited number of longitudinal studies. Still, participants who had periodontal disease and missing teeth had a higher risk of subsequent incident RA (Demmer et al., 2011).

Interestingly, within population genetically at-risk for RA, ACPA-positives individuals have higher prevalence and severity of periodontitis than ACPA-negative subjects (Loutan et al., 2019). The latter suggests a causal role for periodontitis in the development of systemic auto-immunity preceding RA onset. In animal models, periodontitis primed by *Porphyromonas gingivalis* transplantation triggers seropositive arthritis after 4 to 8 months, with systemic inflammation and bone erosions (Courbon et al., 2019). *P gingivalis* has the ability to citrullinate the host’s peptides and produce neo-autoantigens. This observation initially led to the hypothesis that this microbe could be associated with the etiopathogenesis of RA (Li S et al., 2013). Oral *Aggregatibacter actinomycetemcomitans (Aa)* was also later shown to induce *in vivo* hypercitrullination patterns, comparable to those observed in the joints of RA patients (Konig et al., 2016). Moreover at least 47% of patients with established RA were immune for *Aa*, versus only 11% in healthy controls (Konig et al., 2016). However, current evidence does not allow to point out a given bacterial taxa as the unique responsible for oral dysbiosis in RA development. Instead, several inflammatory-associated species, for instance within the *Prevotella* or *Leptotrichia* genus (**Table 1**), associate with RA and periodontitis (Scher et al., 2012; Chen et al., 2018). Overall, this oral dysbiosis tends to attenuate after adequate antirheumatic treatment (Zhang et al., 2015), suggesting that periodontitis might trigger autoimmunity in a subset of patients, but does not necessarily perpetuates the autoimmune process.

**Table 1:** Disease specific microbiota alterations at the species level and associated relevant biological findings.

Disease	Microbiota alteration	Relevant associated biological findings	Future directions ?
Rheumatoid arthritis	**Oral microbiota** -Overall diversity preserved, but different taxa repartition in RA (Mikuls et al., 2018). In particular, *Prevotella* species and *Leptotrichia* species almost absent in healthy controls (Scher et al., 2012; Chen et al., 2018),and depletion of *Haemophilus spp* in RA patients (Zhang et al., 2015; Corrêa et al., 2019). -Infection with *Porphyromonas gingivalis* or *Aggregatibacter actinomycetemcomitans (Aa) (*Konig et al., 2016) more frequent in RA patients.	-Analysis of gingival crevicular fluid from patients with periodontitis revealed extensive protein citrullination with same patterns as observed in RA joints (Konig et al., 2016)-Incubation of neutrophils with *Aa*, but not with *P. gingivalis*, reproduced this hypercitrullination (Konig et al., 2016) **-** Periodontitis status correlates with anti-citrullinated-peptides antibodies (Corrêa et al., 2019).	-Unravel associations between smoking, periodontitis, oral dysbiosis and rheumatoid arthritis onset. -Homogenize sampling methodology across the various oral sites (saliva, crevicular fluid, dental plaque, etc.).
	**Gut microbiota** -Increase of *Prevotella* species, in particular *Prevotella copri*, in early RA and preclinical stages of RA (Scher et al., 2013). **-** *Eggerthella* genus (Zhang et al., 2015; Chen J et al., 2016), and *Collinsella aerofaciens* was also more abundant in some RA patients (Chen J et al., 2016).	- Serological immunity against *P copri* is specific for RA patients (Pianta et al., 2017b). **-** *Prevotella* species have potentially cross-reactive antigens (Pianta et al., 2017a). -Patient-derived *Collinsella aerofaciens* (Chen J et al., 2016), as well as *Prevotella* species aggravate arthritis in mice models.	-Longitudinal follow-up of at-risk population to allow proper causal inference. - Identification of targeted approaches to shape gut microbiota (probiotics, FMT, functional foods, etc.).
Ankylosing spondylitis	**Gut microbiota** -Higher abundance of five families of bacteria *Lachnospiraceae*, *Ruminococcaceae*, *Rikenellaceae*, *Porphyromonadaceae* and *Bacteroidaceae*, and lower abundance of two families of *Veilonellaceae (*Costello et al., 2015b) and *Prevotellaceae*. -A higher prevalence of sulfate-reducing bacteria was also reported in patients with AS.	-A higher prevalence of sulfate-reducing bacteria, also known as hydrogen scavengers. These bacteria can reduce inorganic sulphate to hydrogen sulfide. The higher prevalence of these bacteria may indicate gut inflammation (Stebbings, 2002).	-Modifiye risk factors including nutritional habits (fiber-enriched cereal bars or dietary interventions). - To investigate the effectiveness of fecal transplantation and its long-term results. - Further studies in AS patient’s microbiota needs to focus more on strain level identification of bacteria and plans to study on limitations of 16S-based analysis.
Psoriatic arthritis	-High-throughput 16S rRNA pyrosequencing method in a cohort of PsA and psoriasis patients indicated a decrease in taxonomic diversity due to lower abundances of several taxa compared to the microbiota of healthy controls (Scher et al., 2015), including decreased *Coprococcus* in both psoriasis and PsA patients, while significantly lower levels of *Akkermansia* and *Ruminococcus* only in the PsA (Scher et al., 2015; Scher et al., 2016). - Higher abundance of *Firmicutes* and lower abundance of phylum *Actinobacteria*, *Staphylococci* and *Propionibacteria* in psoriasis patients and in affected skin (Gao et al., 2008; Fahlén et al., 2012; Costello et al., 2015a).	- The decreased abundance of *Ruminococcus* and *Akkermansia* specific to PsA has been evaluated as a chronological loss of diversity that may potentially associate with the natural history of disease (Scher et al., 2015). -The decrease of *Propionibacteria* may be the cause or result of changes in diseased skin. It still remains unknown if it is a potential biomarker to predict who finally improve PsA (Coit and Sawalha, 2016).	-Homogenize sampling methodology across the skin both psoriasis and PsA patients. -Future preventive or treatment strategies could be including microbiota-targeted interventions, such as dietary interventions, probiotics or FMT.
Juvenile spondylarthritis	-Patients with ERA were reported to display decreased abundance of *C. leptum*, similar to AS (Stebbings, 2002; Stoll et al., 2014)., a decrease in *F. prausnitzii* and in cellular immune response to *Salmonella typhimurium* were also reported in patients with juvenile SpA.	-In children with enthesitis-related arthritis (juvenile idiopathic arthritis subtype), the relative abundance of *F. prausnitzii* was presented lower as compared to healthy controls (Stoll et al., 2014).	-Planning of further and larger studies on microbiota analysis of children with ERA. -Education of patients and parents about probiotics and nutritional habits.

### The Gut Microbiota and RA

Early research between the 1970’s and 2000’s already suggested quantitative changes of specific bacterial species in DMARD-naive RA patients, including *Clostridium perfringens (*Olhagen and Månsson, 1968), *Bacteroides*, *Prevotella* and *Porphyromonas* genera (Eerola et al., 1994; Toivanen et al., 2002; Vaahtovuo et al., 2008). After 2010, several studies using 16S RNA/DNA sequencing technology demonstrated an increased relative abundance of *Prevotella copri* in pre-RA or in early RA patients compared to healthy controls (Scher et al., 2013; Maeda et al., 2016; Alpizar-Rodriguez et al., 2019). Interestingly, this relative expansion of *P. copri* was rarely present in established and chronically treated RA patients or in psoriatic arthritis patients (Scher et al., 2013).

In an animal model of arthritis, transplantation of a human microbiota from RA patients dominated by *Prevotella* resulted in severe arthritis, which was not the case when healthy-control-derived microbiota was transplanted (Maeda et al., 2016). The authors have thus postulated that *P. copri* might carry an epitope conferring cross-reactivity to arthritis-related autoantigens. Interestingly, Pianta and al. later isolated a peptide presented by monocytes from a chronic RA patient, peptide belonging to *P. copri* (protein Pc-p27) (Pianta et al., 2017a). Antibody response to this protein or to *P. copri* was detected in 32% of new onset RA patients, while rarely found in other forms of arthritis or in healthy controls (Pianta et al., 2017b). Remarkably, a sequence homology exists between two self-proteins targeted by the immune response in RA (Wang et al., 2017) and epitopes from *Prevotella* species (Pianta et al., 2017a). The latter is suggestive for molecular mimicry and provides a possible mechanism linking intestinal dysbiosis with RA development. However, *P. copri* is certainly not the only intestinal bacteria associated with the pathogenesis of RA. Other microbes, such as *Collinsella aerofaciens*, have also been involved, and proved to worsen arthritis in mice models (Chen J. et al., 2016). Moreover, in established RA patients, dysbiosis with different species have also been described (Zhang et al., 2015; Chen J. et al., 2016). Furthermore, species such as *Prevotella histicola* were even shown to exert a protective effect in mice models (Marietta et al., 2016). Future studies will thus have to better establish the role of intestinal dysbiosis in the development of autoimmunity and the relative contribution of the different bacterial species. Ultimately, we will have to demonstrate that targeting intestinal dysbiosis in individuals at high risk for RA can prevent the onset of the disease.

## Future Directions

Research on the role of microbiota in RMDs has been hampered by the fuzziness of the disease phenotypes studied. The microbiome has been shown to differ between patients with early disease, prior to immunosuppressive therapy, and patients with established and chronically treated disease (Zhang et al., 2015). Moreover, current 16S profiling typically provides only the relative abundance of various species, thus delivering a proportional result, which may be susceptible to biases by extraneous factors, such as bowel habits (Vandeputte et al., 2017). In addition, the IBD literature has suggested a role of the intestinal virome in IBD-specific dysbiosis (Norman et al., 2015) and underlined colitogenic properties of secretory IgA-coated human bacteria (Palm et al., 2014), which may also be relevant in the pathogenesis of RMDs (Stoll, 2015; Scher et al., 2016). The characterization of IgA-coated bacteria in RA population has never been performed.

Overall, there is a need for more quantitative analysis of the microbiome and in-depth longitudinal metagenome-wide association studies addressing the functional capacity of the host microbiota. This will help answer questions that require rigorous exploration such as the directionality (cause or consequence of autoimmune process) of the dysbiotic process. The timing of microbial perturbation in the clinical course of the disease remains unclear. The contribution of communities in specific body niches (i.e. the periodontal and lung and the skin in PsA) and the potential utility of the microbiome in pharmacokinetics of immunosuppressive therapy are also of interest (Coit and Sawalha, 2016; Scher et al., 2016). Future microbiome research in RMDs needs to focus more on strain level identification of bacteria and overcome limitations of 16S-based analyses, which might not be precise enough. For example, Palm et al. could demonstrate that bacterial isolates from different IBD patients that were taxonomically assigned as the same species *via* 16S sequencing often displayed differential IgA-coating patterns, corresponding to genetically distinct strains and very different pathological capabilities (Palm et al., 2014).

A therapeutic window of opportunity for anti-rheumatic therapy exists in the early phases of RMDs (Finckh et al., 2006), and it is widely believed that an even earlier intervention, prior to the development of arthritis, could be curative (Finckh and Deane, 2014). In humans, at least six different randomized controlled trials are testing the efficacy of preventive medicinal approaches (Finckh and Deane, 2014). Nonetheless, people at high risk for RMDs appear to prefer non-medicinal interventions, such as dietary interventions, stress reduction or physical exercise (Novotny et al., 2013). Therefore, studies are exploring the efficacy of modifying risk factors of RMDs, including nutritional habits (Sparks et al., 2014), change in oral microbiome by treating periodontal disease (University Hospital, Toulouse, 2018) or by administering probiotics (Alipour et al., 2014; Wang et al., 2016). For instance, a double blinded placebo-controlled trial showed that daily supplementation of RA patients with *Lactobacillus casei 01* improved disease activity after 8 weeks (Vaghef-Mehrabany et al., 2014). Nevertheless, other studies with different *Lactobacillus* strains were inconclusive (de los Angeles Pineda et al., 2011). Overall, a meta-analysis published in 2017 concluded that even though probiotics decreased inflammatory cytokines, clinical effect in established RA was still unclear (Mohammed et al., 2017). Whether these conclusions apply to at-risk population, as a preventive measure prior to disease occurrence, has not been studied yet.

Another potentially attractive intervention for intestinal dysbiosis is fecal microbiota transplantation (FMT), which is defined as the administration of fecal micro-organisms from a healthy donor into the digestive tract of a recipient patient. FMT is a successful treatment for *Clostridium difficile* infections (Surawicz et al., 2013; Cammarota et al., 2017), and is currently tested in immune mediated diseases, such as ulcerative colitis (Moayyedi et al., 2015; Rossen et al., 2015a; Paramsothy et al., 2017). It is indeed considered to be a safe procedure (Rossen et al., 2015b; Cohen and Maharshak, 2017), even in debilitated or immunosuppressed patients (Agrawal et al., 2016; Friedman-Korn et al., 2018) although long-term side effects are largely unknown. No study has yet established the feasibility and safety of FMT in RMDs. However, studies with FMT are ongoing in psoriatic arthritis patients (Kragsnaes et al., 2018) and RA patients. Functional foods, such as fiber-enriched cereal bars (Häger et al., 2019), or dietary interventions, could also be considered as future tools for shaping intestinal flora, even though solid evidence in this field is still lacking.

## Conclusion

The direct and indirect interplay between the microbiome and the host immune responses is now increasingly appreciated, as more human microbiome studies become available and the mucosal immunity is better understood (Scher et al., 2016; Caminer et al., 2017). High-throughput sequencing has become a valuable tool, with the potential to offer a new biologic basis for classification and clues for pathogenesis in RMDs (Gill et al., 2015; Coit and Sawalha, 2016; Scher et al., 2016). So far, various dysbiosis have been identified in the microbiota of patients with specific RMDs compared to controls (Alpizar-Rodriguez et al., Stebbings, 2002; Scher et al., 2013; Stoll et al., 2014; Costello et al., 2015b; Gill et al., 2015; Scher et al., 2015; Maeda et al., 2016). Furthermore, gut microbiota from diseased patients has been shown to trigger or exacerbate disease phenotypes in RMDs mouse models (Šinkorová et al., 2008; Ruutu et al., 2012; Sherlock et al., 2012; Maeda et al., 2016). Etiopathogenic hypotheses generally involve a microbiota related activation of intestinal Th9 (Ciccia et al., 2016), Th17 cells (DeLay et al., 2009; Rosenbaum and Davey, 2011; Glatigny et al., 2012), or molecular mimicry with mucosal bacteria (Pianta et al., 2017a; Pianta et al., 2017b; Wang et al., 2017). However, many more questions remain to be addressed before a causal relationship of the human microbiome in the pathogenesis of rheumatic diseases is definitely established. The specific role of the microbiota in the development of RMDs and exact immunological mechanisms need to be better understood (Gill et al., 2015; Scher et al., 2016; Coit and Sawalha, 2016), while methodology needs to be harmonized and updated. If the findings of this line of research hold true, the translation into clinical practice would result in novel preventive or curative strategies, which could include microbiota-targeted interventions, such as dietary modifications, functional foods, probiotics, or FMT.

## Author Contributions

MV, BG, IÜ, SC, and AF contributed to the design and implementation of the review and to the writing of the manuscript. All authors contributed to the article and approved the submitted version.

## Conflict of Interest

The authors declare that the research was conducted in the absence of any commercial or financial relationships that could be construed as a potential conflict of interest.

## Abbreviations

AS, ankylosing spondylitis; FMT, fecal microbiota transplantation; IBD, inflammatory bowel disease; PsA, psoriatic arthritis; RA, rheumatoid arthritis; ReA, reactive arthritis; SpA, spondylarthritis.
